# Supplementary material for: The Anti-Arthritic Potential of the Ethanolic Extract of Salvia Lachnostachys Benth. Leaves and Icetexane Dinor-Diterpenoid Fruticuline B
Source: Pharmaceuticals (Basel). 2024 Sep 18;17(9):1226. doi: 10.3390/ph17091226 (PMC11435285; doi:10.3390/ph17091226)
Supplement: Supplementary file 1 [file pharmaceuticals-17-01226-s001.zip › pharmaceuticals-3148695-supplementary.pdf]

Supplementary Material

The potential anti-arthritic effects of the ethanolic extract of *Salvia lachnostachys* Benth. leaves and icetexane *dinor*-diterpenoid Fruticuline B

Natália de M. Balsalobre[1†], Elisangela dos Santos-Procopio[1†], Cristhian S. Oliveira[2], Silvia C. Neves[3], Maria H. Verdan[4], Saulo E. Silva-Filho[5], Rodrigo J. Oliveira[3], Maria É. A. Stefanello[2], Cândida A. L. Kassuya[1*]

[1]Federal University of Grande Dourados, Faculty of Health Sciences, 79804-970, Dourados, MS, Brazil.
[2]Federal University of Paraná, Chemistry Department, 81530-900, Curitiba, PR, Brazil.
[3]Federal University of Mato Grosso do Sul, Faculty of Health, CeTroGen, 79070-900, Campo Grande, MS, Brazil.
[4]Federal University of Grande Dourados, Postgraduate Program in Chemistry, 79804-970, Dourados, MS, Brazil.
[5]Federal University of Mato Grosso do Sul, Faculty of Pharmaceutical Sciences, Food and Nutrition, 79070-900, Campo Grande, MS, Brazil.

†These authors contributed equally to this work.

*Author for correspondence: Candida Aparecida Leite Kassuya (candida2005@gmail.com), Phone: +55 67 3410-2326, Fax: +55 67 3410-2326.

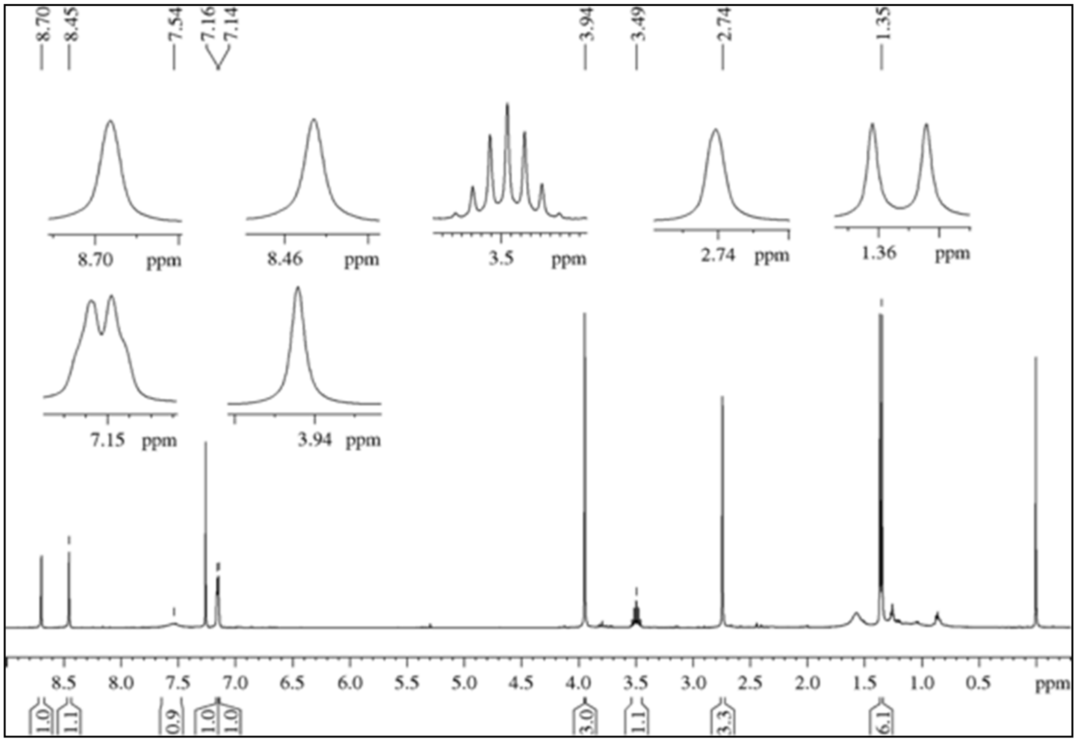

Figure S1. ^{1}H NMR spectrum (400 MHz, $CDCl_3$) of fruticuline B.

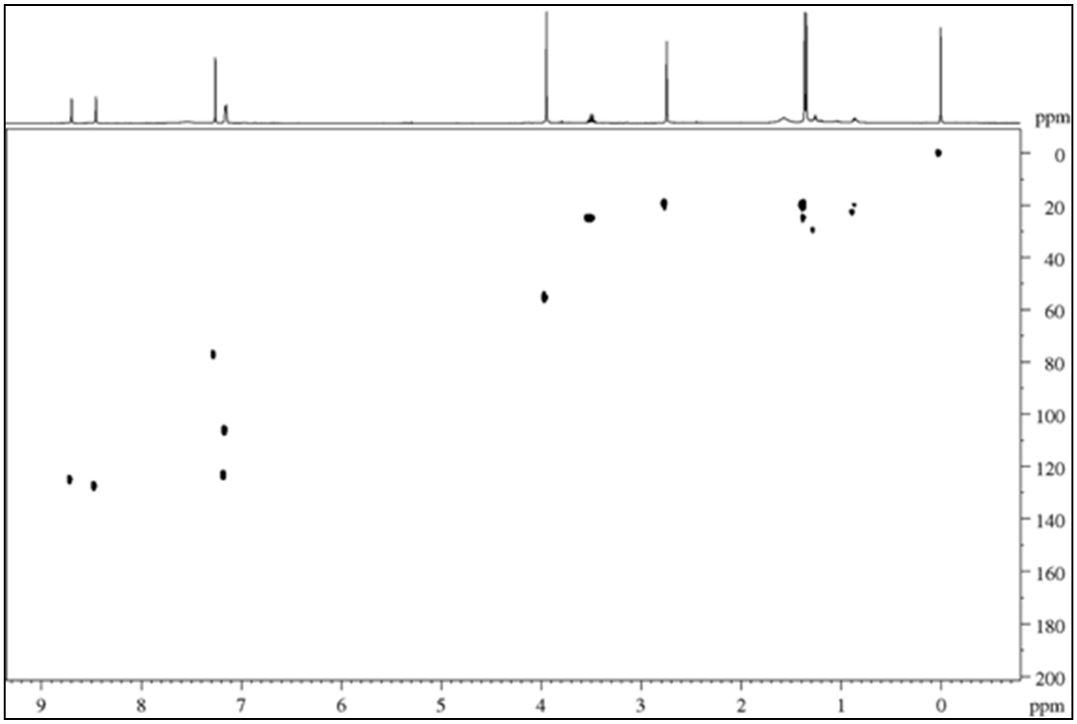

Figure S2. HSQC spectrum (400 MHz, $CDCl_3$) of fruticuline B.

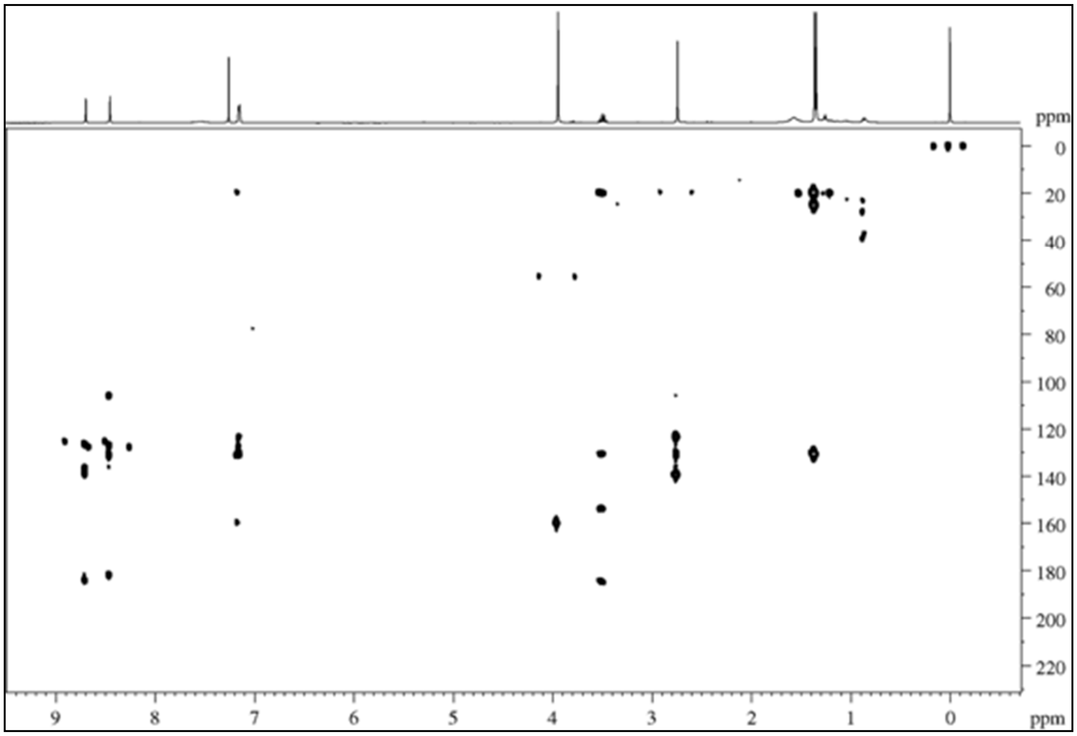

Figure S3. HMBC spectrum (400 MHz, $CDCl_3$) of fruticuline B.
